# Potential association with malnutrition and allocation of combination medical therapies in hospitalized heart failure patients with reduced ejection fraction

**DOI:** 10.1038/s41598-022-12357-4

**Published:** 2022-05-18

**Authors:** Yumiko Kawakubo, Yasuyuki Shiraishi, Shun Kohsaka, Takashi Kohno, Ayumi Goda, Yuji Nagatomo, Yosuke Nishihata, Mike Saji, Makoto Takei, Yukinori Ikegami, Nozomi Niimi, Alexander Tarlochan Singh Sandhu, Shintaro Nakano, Tsutomu Yoshikawa, Keiichi Fukuda, West Tokyo Heart Failure Registry, Yasuyuki Shiraishi, Shun Kohsaka, Takashi Kohno, Ayumi Goda, Yuji Nagatomo, Yosuke Nishihata, Mike Saji, Makoto Takei, Yukinori Ikegami, Shintaro Nakano, Tsutomu Yoshikawa

**Affiliations:** 1 Division of Cardiology, Department of Medicine, Keio University School of Medicine, 35 Shinanomachi Shinjuku-ku, Tokyo, 160-8582 Japan; 2 Department of Cardiovascular Medicine, Kyorin University Faculty of Medicine, Tokyo, Japan; 3 Department of Cardiology, National Defense Medical College Hospital, Tokorozawa, Japan; 4 Department of Cardiology, St. Luke’s International Hospital, Tokyo, Japan; 5 Department of Cardiology, Sakakibara Heart Institute, Tokyo, Japan; 6 Department of Cardiology, Saiseikai Central Hospital, Tokyo, Japan; 7 Department of Cardiology, National Hospital Organization Tokyo Medical Center, Tokyo, Japan; 8 Department of Cardiovascular Medicine, Stanford University, Stanford, CA USA; 9 Department of Cardiology, Saitama Medical University International Medical Center, Saitama, Japan

**Keywords:** Cardiology, Heart failure

## Abstract

Malnutrition is common in patients with heart failure with reduced ejection fraction (HFrEF) and may influence the long-term prognosis and allocation of combination medical therapy. We reviewed 1231 consecutive patient-level records from a multicenter Japanese registry of hospitalized HFrEF patients. Nutritional status was assessed using geriatric nutritional risk index (GNRI). Combination medical therapy were categorized based on the use of beta-blockers, renin-angiotensin system inhibitors, and mineralocorticoid receptor antagonists. The composite outcome of all-cause death and HF rehospitalization was assessed. The mean age was 72.0 ± 14.2 years and 42.6% patients were malnourished (GNRI < 92). At discharge, 43.6% and 33.4% of patients were receiving two and three agents, respectively. Malnourished patients had lower rates of combination medical therapy use. The standardized GNRI score was independently associated with the occurrence of adverse events (hazard ratio [HR]: 0.88, 95% confidence interval [CI] 0.79–0.98). Regardless of the GNRI score, referenced to patients receiving single agent, risk of adverse events were lower with those receiving three (HR: 0.70, 95% CI 0.55–0.91) or two agents (HR: 0.70, 95% CI 0.56–0.89). Malnutrition assessed by GNRI score predicts long-term adverse outcomes among hospitalized HFrEF patients. However, its prognosis may be modified with combination medical therapy.

## Introduction

Heart failure (HF) with reduced ejection fraction (HFrEF; left ventricular ejection fraction [LVEF] of ≤ 40%) affects over 2.5 million adults in the United States alone and is associated with high economic costs and rates of impaired health status, morbidity, and mortality^1–3^. Similar trends are observed in Japan^4^, where more than 230,000 patients were hospitalized for HF in 2015. This number appears to be increasing by more than 10,000 cases each year, with one-half of these cases involving HFrEF^5^. High-quality evidence currently supports the treatment of HFrEF patients using combination medical therapy, which has traditionally included beta-blockers (BBs), angiotensin-converting enzyme inhibitors (ACEis)/angiotensin receptor blockers (ARBs), and mineralocorticoid receptor antagonists (MRAs)^5–7^. Novel disease-modifying therapies, such as angiotensin receptor-neprilysin inhibitors (ARNIs) and sodium-glucose cotransporter-2 inhibitors (SGLT2is), have also been introduced, and these agents are expected to further improve the outcomes of HFrEF patients incremental to the backbone of combination medical therapy listed above^8^.

Previous studies have identified important barriers to the full implementation of combination medical therapy, including renal dysfunction, hyperkalemia, hypotension, bradycardia, old age, and low functional status^9–11^. Malnutrition is also common among patients with HF and is independently associated with adverse outcomes, which are linked to both HF and mechanisms that are related to other chronic disease states^12^. In addition, malnutrition may reflect a state of abnormal drug metabolism, which may cause unfavorable treatment effects^13,14^. Therefore, malnutrition can also negatively affect clinical decision-making for patients with significant clinical conditions, such as cancer^15,16^. However, contemporary large-scale randomized controlled trials (RCTs) have generally excluded malnourished patients, and there is limited data regarding the interaction between nutritional status and real-world use of HF-related combination medical therapy^17,18^. Given the typically stringent entry criteria for clinical trials, observational studies may provide a more realistic assessment of malnutrition among the HF patients.

This study evaluated the prevalence of malnutrition among HFrEF patients, their long-term outcomes, and whether their nutritional status was associated with the use of combination medical therapy. The West Tokyo Heart Failure (WET-HF) registry in Japan has historically included HF patients who have lower body mass index or who are older (vs. Western HF studies^19^), thereby providing a unique opportunity to investigate this topic.

## Methods

### Study design

Patient-level data were extracted from the WET-HF registry^20,21^, which prospectively collected information on patients who were admitted to three university hospitals and three tertiary referral hospitals within the Tokyo metropolitan area (Japan) in 2006–2017. This multicenter registry was designed to collect data on the clinical characteristics and outcomes of patients hospitalized with a primary diagnosis of acute HF, which is defined as rapid-onset HF with changes in signs and symptoms that indicate an urgent need for hospitalization and therapy^22^. Clinical diagnoses were made by experienced cardiologists at each institution, who excluded patients who presented with acute coronary syndrome. A robust assessment of care and patient outcomes was facilitated by dedicated clinical research coordinators collecting baseline data and outcomes from medical records and querying attending physicians. Data were entered into an electronic data-capturing system that has a robust data query engine and system validations to ensure data quality. Exclusive on-site auditing by the investigators ensured proper registration of each patient. This study’s retrospective protocol was approved by the Institutional Review Board of Keio University School of Medicine and conducted in accordance with the Declaration of Helsinki. And all patients provided informed consent for their treatment and research use of their data.

### Patient population

**Figure 1:**
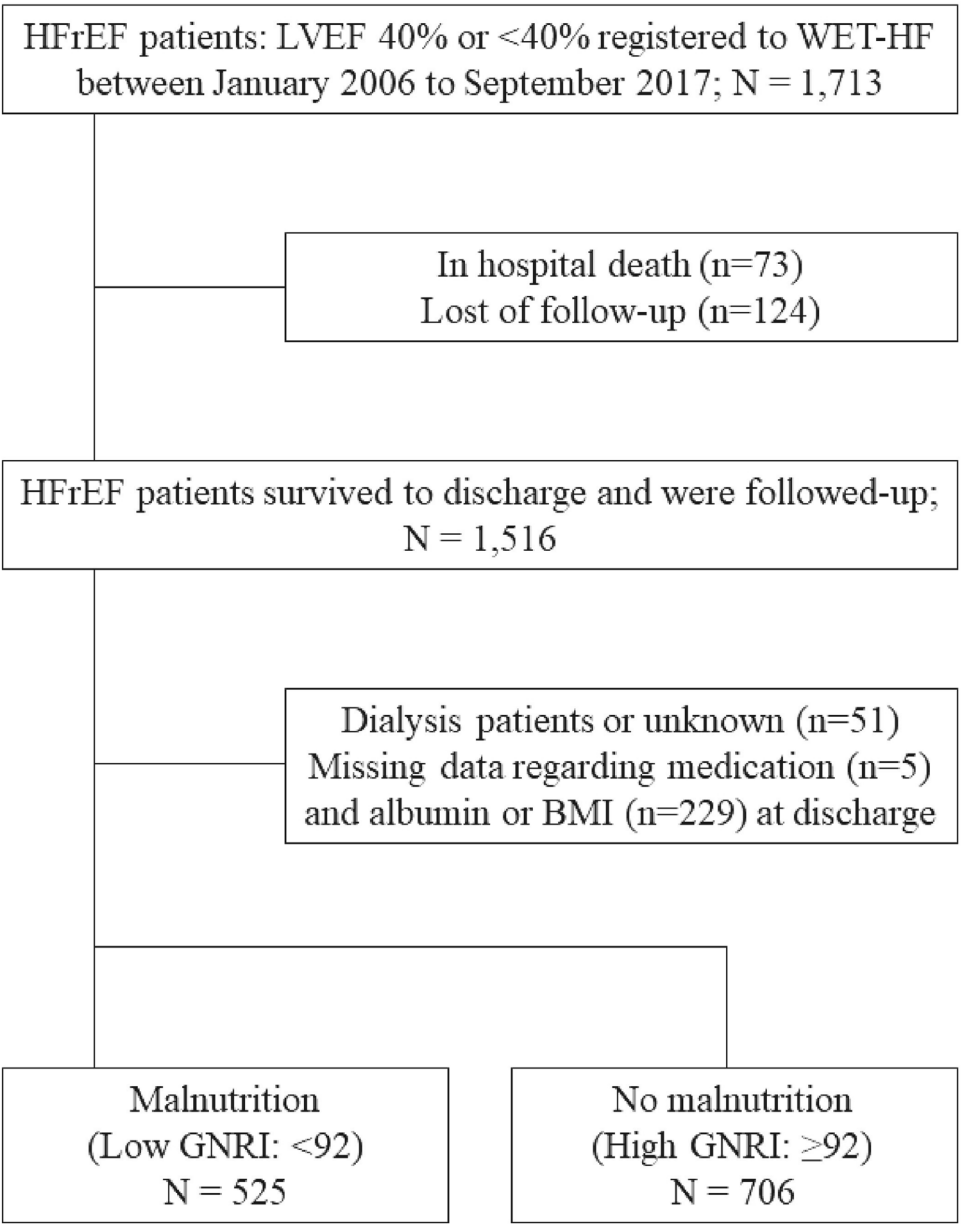
Study flowchart. The HFrEF patients were categorized according to their GNRI score into groups with malnutrition (low GNRI) or normal nutritional status (high GNRI). *HFrEF* heart failure with reduced ejection fraction, *WET-HF* West Tokyo Heart Failure, *GNRI* geriatric nutritional risk index.

This study evaluated prospectively collected data from 1713 consecutive patients who were hospitalized for HFrEF. However, it excluded 73 patients who died during hospitalization, 51 dialysis patients, 124 patients who were lost to follow-up, 229 patients with missing albumin or body mass index data that prevented evaluation of nutritional status, and five patients with missing data regarding medication use at discharge. Thus, the study analyzed the complete data from 1231 patients (Fig. 1).

### Definitions of variables and outcomes

Baseline characteristics included age, sex, causes of HF, medical history, previous procedures, vital signs, laboratory data, echocardiographic data, and medications used at discharge. The definition of a reduced ejection fraction (EF ≤ 40%) was based on the universal definition and classification of HF^23^.

Combination medical therapy, or optimal medical therapy for single agent use, was defined as the prescription of BBs, RASis (ACEis or ARBs), and MRAs at the time of discharge. During the study period, ARNI, SGLT2i, and ivabradine for HF patients were not available in Japan. Thus, based on the medication(s) at discharge, we classified the patients as having received none of the three agents, single therapy (one agent), double therapy (two agents), or triple therapy (all three agents).

Patients at risk of malnutrition can be identified using several risk indexes. The present study evaluated nutritional status based on the GNRI, which is a simple formula that has been demonstrated to be clinically useful among patients with various medical conditions^24,25^. The GNRI score was calculated at the time of discharge as follows:

$$14.89 \times {\text{serum}}\,{\text{albumin}}\,\,({\text{g}}/{\text{dL}}) + 41.7 \times {\text{body}}\,{\text{mass}}\,{\text{index}}\,({\text{kg}}/{\text{m}}^{2} )/22$$

Clinical characteristics and mortality were compared between the following two groups: low GNRI group (< 92) with moderate or severe nutritional risk and high GNRI group (≥ 92) with low or no nutritional risk according to a previous report^26^. In the present study, patients with low GNRI score were defined as “malnourished.”

Ischemic etiology was defined as left ventricular dysfunction (EF of ≤ 40%) with a history of myocardial infarction, percutaneous coronary intervention, coronary artery bypass grafting, or at least one major epicardial coronary artery with ≥ 75% stenosis. As coronary angiography (CAG) is usually recommended for patients with a high pre-test probability of coronary artery disease, CAG was performed during index hospitalization for selected patients based on their clinical course, in this cohort. The eGFR was calculated using the Modification of Diet in Renal Disease Equation for Japanese Patients, which was proposed by the Japanese Society of Nephrology^27^. The EF was calculated using the modified Simpson’s method. Ultrasound cardiography was performed by highly experienced cardiologists or clinical technologists during the hospitalization. The NYHA functional class was evaluated at admission by the treating cardiologists at each institution.

Follow-up data were mainly collected via telephone contact or a chart review. The primary outcome of interest was defined as a composite of all-cause death and HF rehospitalization after discharge, with the decision to rehospitalize the patient made by the treating physicians based on the usual standard of care. Each of these events was assessed individually as secondary endpoints.

To ensure the accuracy of the adverse event ascertainment, the WET-HF registry is supported by a central study committee that adjudicates determination of the endpoint.

### Statistical analysis

Data were reported as number and percentage for categorical variables and as median (interquartile range) for continuous variables. Continuous variables were compared using the Mann–Whitney U test and Kruskal–Wallis test, whereas categorical variables were compared using the Pearson’s chi-squared test.

A multivariable logistic regression model was used to evaluate whether medical therapy use was related to different clinical variables. Models were adjusted for age, sex, serum potassium concentration, total cholesterol level, eGFR level, LVEF, systolic blood pressure, heart rate, history of HF hospitalization, atrial fibrillation, hypertension, diabetes mellitus, chronic obstructive pulmonary disease, ischemic cardiomyopathy, and GNRI status. Survival curves were compared using the Kaplan–Meier method and log-rank test.

Multivariable Cox proportional hazard models were also created to assess the association between combination medical therapy use (none, single, double, or triple therapy) and each endpoint, which was adjusted for age, sex, systolic blood pressure, and heart rate at discharge, renal dysfunction (eGFR < 60 mL/min/1.73 m^2^), LVEF, history of HF hospitalization, ischemic etiology, atrial fibrillation, chronic obstructive pulmonary disease, stroke, diabetes mellitus, use of loop diuretics, statin use, and standardized GNRI score. Results were reported as HRs, 95% CIs, and p-values. We also created a 4-knot restricted cubic spline model of the HRs according to the GNRI status (continuous value), which was adjusted for the same factors as the Cox hazards model.

A sensitivity analysis was also performed after excluding patients with advanced renal impairment (eGFR < 30 mL/min/1.73 m^2^) or higher mortality risk (an estimated mortality risk of > 10% based on a GWTG-HF risk score of > 57), considering the possibility of patients who may not benefit from or tolerate combination medical therapy^21^. To verify the GNRI cutoff points, we performed a sensitivity analysis using the median of the GNRI (< or ≥ 94.1) in the whole analysis cohort. All analyses were performed at a two-sided significance level of 0.05. There was no correction for multiple comparisons given the exploratory nature of this observational analysis. IBM SPSS software (version 26; IBM Corp., Armonk, NY, USA) and R software (version 3.6.3; Foundation for Statistical Computing, Vienna, Austria) were used for statistical analysis.

### Ethics approval and participant’s consent

The study’s retrospective protocol was approved by the Institutional Review Board of Keio University School of Medicine and conducted in accordance with the Declaration of Helsinki. And all patients provided informed consent for their treatment and research use of their data.

## Results

### Baseline characteristics

The 1231 HFrEF patients were predominantly men (62%) and had a median age of 72.0 years (interquartile range: 61.0–81.0 years). The baseline characteristics of patients divided into two groups according to their GNRI values are listed in Table 1. The low GNRI group had a higher proportion of female patients, tended to be older, had more severe symptoms (New York Heart Association [NYHA] III or IV), had lower hemoglobin and albumin concentrations, and had higher creatinine and B-type natriuretic peptide concentrations. There were no significant inter-group differences in terms of systolic blood pressure and heart rate at admission. At the time of discharge, 20, (IQR 20–40) mg/day of loop diuretic (converted to furosemide dose) and 25% (IQR 12.5–50) of maximum dose of beta-blocker (converted to carvedilol dose) were prescribed.

**Table 1:** Baseline characteristics of patients by nutritional status.

Variables	Low GNRI (< 92) (n = 525)	High GNRI (GNRI ≥ 92) (n = 706)	p value
Age, years	78 [69, 84]	68 [57, 77]	< 0.001
Female, n (%)	200 (38.1)	159 (22.5%)	< 0.001
Systolic blood pressure, mmHg	132 [110, 151]	132 [112, 155]	0.507
Heart rate, beats/min	94 [78, 114]	96 [78, 112]	0.814
LVEF, %	30 [25, 35]	30 [23, 35]	0.013
Ischemic etiology, n (%)	205 (39.0)	246 (34.8)	0.130
NYHA (III–IV)	432 (86.7)	553 (81.4)	0.015
**Comorbidities**			
Prior HF hospitalization, n (%)	188 (36.4)	246 (35.1)	0.642
Hypertension, n (%)	342 (65.1)	440 (62.3)	0.309
Diabetes mellitus, n (%)	201 (38.3)	270 (38.2)	0.988
Dyslipidemia, n (%)	193 (37.3)	319 (45.8)	0.003
Smoking, n (%)	209 (41.4)	357 (52.2)	< 0.001
Atrial fibrillation, n (%)	201 (38.4)	290 (41.1)	0.336
Stroke, n (%)	70 (13.3)	89 (12.7)	0.728
COPD, n (%)	26 (5.0)	24 (3.4)	0.173
**Laboratory findings at admission**			
Hemoglobin, g/dL	11.9 [10.4, 13.5]	13.4 [12.0, 15.0]	0.001
BUN, mg/dL	25.1 [18.2, 36.3]	20.3 [15.9, 27.4]	< 0.001
Creatinine, mg/dL	1.14 [0.81, 1.61]	1.04 [0.86, 1.31]	0.003
eGFR, ml/min/1.73 m^2^	45.2 [29.8, 61.6]	53.6 [40.2, 66.5]	< 0.001
Sodium, mEq/L	139 [137, 142]	140 [138, 142]	< 0.001
Potassium, mEq/L	4.4 [3.9, 4.8]	4.3 [4.0, 4.7]	0.823
BNP, pg/mL	1155 [702, 1989]	707 [383, 1082]	< 0.001
Alb, mg/dL	3.4 [3.1, 3.7]	3.8 [3.6, 4.1]	< 0.001

Values are median [with interquartile ranges] or n (%).

*GNRI* geriatric nutritional risk index, *NYHA* New York Heart Association, *LVEF* left ventricular ejection fraction, *HF* heart failure, *COPD* chronic obstructive pulmonary disease, *BUN* blood urea nitrogen, *eGFR* estimated glomerular filtration rate, *BNP* B-type natriuretic peptide.

### GNRI and outcomes

**Figure 2:**
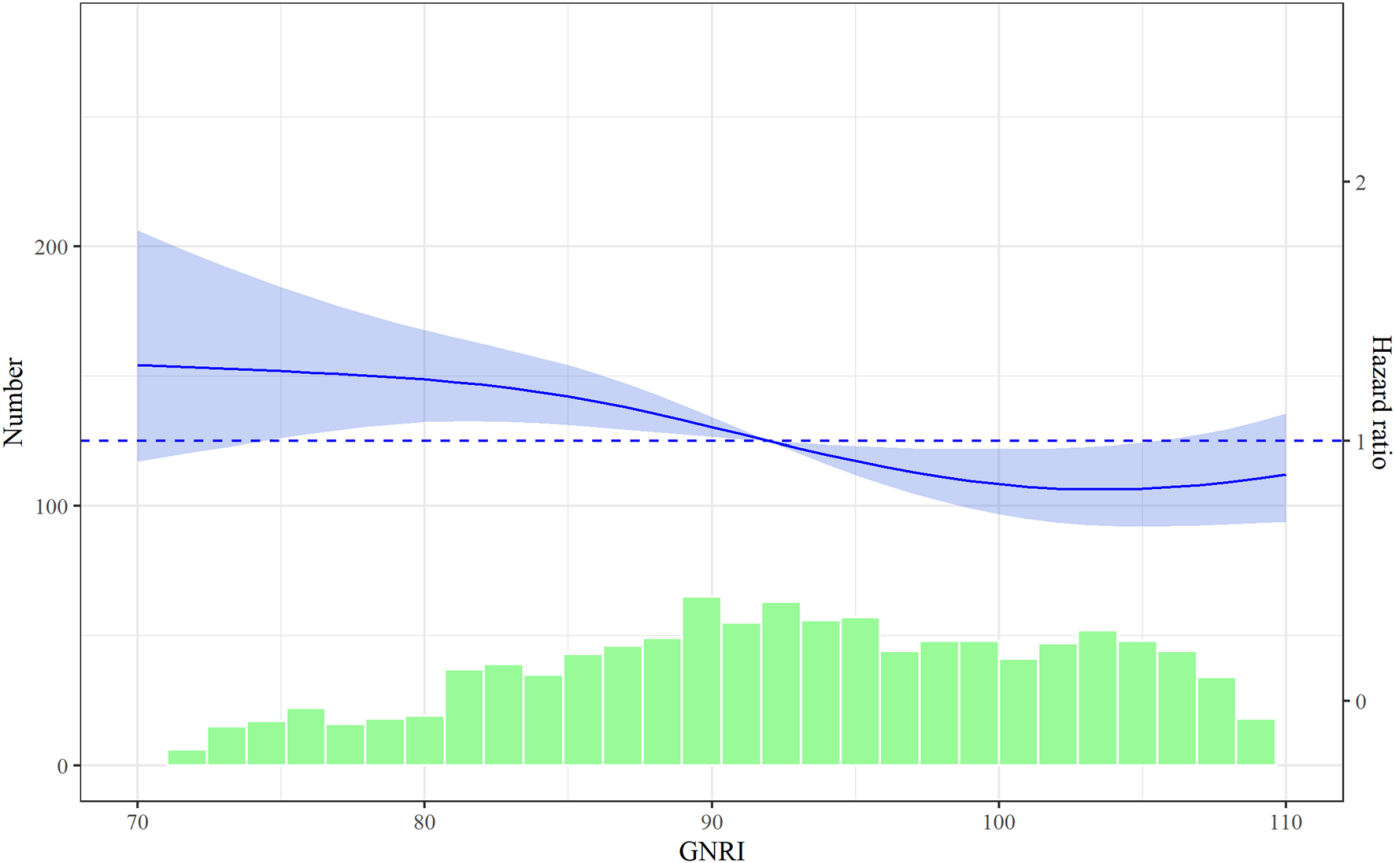
Hazard ratios for the composite outcome (all-cause death and heart failure rehospitalization) according to the Geriatric Nutritional Risk Index score. The blue line represents the continuous hazard ratio (HRs) and the light blue area represents the 95% confidence intervals. The green bars represent the numbers of patients with each Geriatric Nutritional Risk Index (GNRI) score.

During a median follow-up period of 2.0 years (interquartile range: 0.8–3.1 years), the composite outcome was observed for 549 patients (44.6%), including 298 patients (24.3%) who died and 414 patients (33.6%) who were re-hospitalized because of HF. The unadjusted Kaplan–Meier curves revealed that the low GNRI group had a higher risk of the composite outcome (Supplementary Fig. S1). The restricted cubic spline analysis also revealed a positive correlation between the GNRI score and long-term outcomes, particularly among patients with a GNRI score of < 100 (Fig. 2).

### Factors influencing the implementation of optimal medical therapy

At discharge, 49 patients (4.0%) were receiving none of the three agents, 234 patients (19.0%) were receiving a single agent, 537 patients (43.6%) were receiving double therapy, and 411 patients (33.4%) were receiving triple therapy. The overall usage rates were 87.4% for BBs, 71.7% for RASis, and 51.0% for MRAs (Supplementary Fig. S2). The triple therapy group was younger, had a higher frequency of non-ischemic dilated cardiomyopathy, and had better renal function. Furthermore, the combination medical therapy group had a significantly higher median GNRI score (triple therapy: 96.7 [interquartile range: 88.5–104.8], double therapy: 94.5 [interquartile range: 87.3–102.9], single therapy: 91.2 [interquartile range: 82.3–98.2], and no therapy: 88.6 [interquartile range: 82.1–94.8]; p < 0.001).

**Figure 3:**
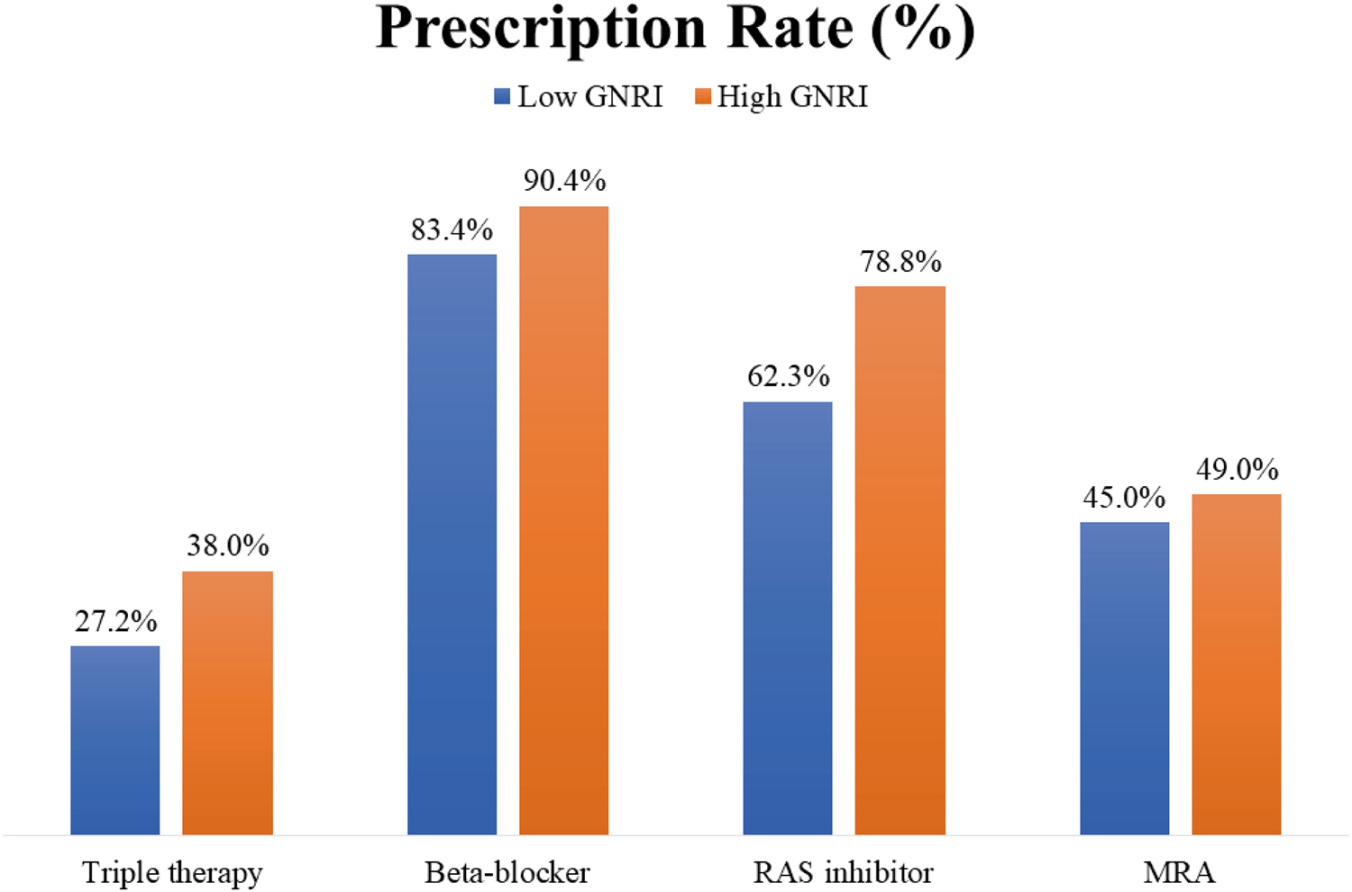
Prescription rate of each agent according to Geriatric Nutritional Risk Index: Low GNRI for moderate or severe nutritional risk with GNRI < 92, and high GNRI for low or no nutritional risk with GNRI ≥ 92. *GNRI* geriatric nutritional risk index, *RASi* renin-angiotensin-system inhibitor, *MRA* mineralocorticoid receptor antagonist, *GNRI* geriatric nutritional risk index, *ACEi* angiotensin-converting enzyme inhibitor, *ARB* angiotensin receptor blocker, *MRA* mineralocorticoid receptor antagonist.

The triple therapy as well as specific therapy groups (i.e., the BB, RASi, and MRA groups) were compared according to their nutritional risk using GNRI values. Lower GNRI values tended to be associated with lower administration rates of triple therapy and each agent (Fig. 3). The associations between specific therapy groups and clinical factors are described in Table 2. After multivariable adjustment, age was significantly associated with BB use (OR: 0.95, 95% confidence interval [CI] 0.93–0.97; p < 0.001) and renal function was associated with RASi use (OR: 1.02, 95% CI 1.02–1.03; p < 0.001) and MRA use (OR: 1.01, 95% CI 1.00–1.02; p = 0.004). Furthermore, the GNRI score was significantly associated with BB use (OR: 1.02, 95% CI 1.00–1.04; p = 0.043) and RASi use (OR: 1.03, 95% CI 1.02–1.05; p < 0.001), but not with MRA use (OR: 1.00, 95% CI 0.99–1.01; p = 0.597).

**Table 2:** Clinical variables contributing to optimal medial therapy administration.

Variable	Beta-blockers		RAS-inhibitors		MRAs	
	OR [95% CI]	p value	OR [95% CI]	p value	OR [95% CI]	p value
Male	0.57 [0.37–0.90]	0.016	0.84 [0.60–1.16]	0.287	0.83 [0.62–1.11]	0.209
Age (per 1 year increase)	0.95 [0.93–0.97]	< 0.001	0.99 [0.98–1.00]	0.164	0.99 [0.98–1.01]	0.234
Prior HF hospitalization	1.06 [0.69–1.62]	0.805	1.12 [0.82–1.54]	0.473	1.61 [1.22–2.13]	< 0.001
Systolic blood pressure (per 1 mmHg increase)	1.00 [1.00–1.01]	0.512	1.00 [1.00–1.01]	0.202	0.99 [0.99–1.00]	0.310
Heart rate (per 1 beat/min increase)	1.01 [1.00–1.01]	0.171	1.00 [0.99–1.00]	0.149	1.00 [0.99–1.01]	0.469
eGFR (per 1 mL/min/1.72 m^2^ increase)	1.00 [0.99–1.01]	0.912	1.02 [1.02–1.03]	< 0.001	1.01 [1.00–1.02]	0.004
Potassium level (per 1 mEq/L increase)	1.32 [0.88–1.98]	0.188	1.15 [0.86–1.54]	0.361	0.95 [0.73–1.24]	0.721
Total cholesterol level (per 1 mg/dL)	1.00 [0.99–1.00]	0.239	1.00 [1.00–1.01]	0.544	1.00 [1.00–1.00]	0.034
Atrial fibrillation	0.97 [0.65–1.46]	0.882	1.20 [0.88–1.64]	0.247	0.92 [0.70–1.20]	0.528
Ischemic cardiomyopathy	1.17 [0.77–1.77]	0.456	1.23 [0.90–1.68]	0.200	1.15 [0.87–1.51]	0.339
Diabetes mellitus	1.02 [0.67–1.54]	0.946	0.78 [0.58–1.06]	0.782	1.20 [0.92–1.57]	0.178
Hypertension	1.43 [0.94–2.16]	0.094	1.36 [0.99–1.88]	0.060	0.99 [0.75–1.31]	0.948
COPD	0.61 [0.28–1.29]	0.228	1.41 [0.66–2.97]	0.374	0.67 [0.35–1.28]	0.228
LVEF (per 1% increase)	0.98 [0.95–1.01]	0.103	1.00 [0.98–1.02]	0.855	0.97 [0.95–0.99]	0.002
GNRI (continuous variable)	1.02 [1.00–1.04]	0.043	1.03 [1.02–1.05]	< 0.001	1.00 [0.99–1.01]	0.597

*RAS-I* renin-angiotensin system inhibitor, *MRA* mineralocorticoid receptor antagonist, *HF* heart failure, *eGFR* estimated glomerular filtration rate, *COPD* chronic obstructive pulmonary disease, *LVEF* left ventricular ejection fraction, *GNRI* geriatric nutritional risk index.

### Implementation of combination medical therapy and clinical outcomes

The unadjusted Kaplan–Meier curves revealed that patients who received more therapeutic agents had a lower risk of the composite outcomes (Supplementary Fig. S3). The multivariable Cox proportional hazards model revealed that, regardless of the GNRI status, combination medical therapy was significantly associated with a lower risk of the composite outcome after adjustment for other patient characteristics. Compared with patients who received single therapy, the risk of the composite outcome was higher among those receiving none of the three agents (adjusted hazard ratio [HR]: 1.62, 95% CI 1.07–2.47; p = 0.024) and lower among those receiving double therapy (adjusted HR: 0.70, 95% CI 0.56–0.89; p = 0.003) and triple therapy (adjusted hazard ratio [HR]: 0.70, 95% CI 0.55–0.91; p = 0.006) (Table 3). Furthermore, the effects of combination medical therapy appeared to be consistent in lowering the risks of all-cause death (triple therapy: HR: 0.67, 95% CI 0.46–0.96; p = 0.028 or double therapy: HR: 0.69, 95% CI 0.50–0.96; p = 0.028) (Supplementary Fig. S4), and hospitalization for HF (triple therapy: HR: 0.65, 95% CI 0.49–0.87; p = 0.003 or double therapy: HR: 0.67, 95% CI 0.52–0.88; p = 0.004) (Supplementary Fig. S5), respectively.

**Table 3:** Multivariable Cox proportional hazards models of the primary outcome.

Medication class	Outcome analysis	
	HR [95%CI]	p value
Triple therapy	0.70 [0.55–0.91]	0.006
Double therapy	0.70 [0.56–0.89]	0.003
Single therapy	Reference	
None medical therapy	1.62 [1.07–2.47]	0.024

These models were adjusted by the following variables: age, sex, systolic blood pressure, heart rate, renal dysfunction (eGFR < 60 ml/min/1.73m^2^), ejection fraction, history of heart failure hospitalization, ischemic etiology, atrial fibrillation, chronic obstructive pulmonary disease, stroke, diabetes mellitus, use of loop diuretics, use of statins and medical therapy (triple, double, single, and no optimal medical therapy), as well as geriatric nutritional risk index.

*BB* beta-blocker, *RASi* renin-angiotensin system inhibitor, *MRA* mineralocorticoid receptor antagonist.

**Figure 4:**
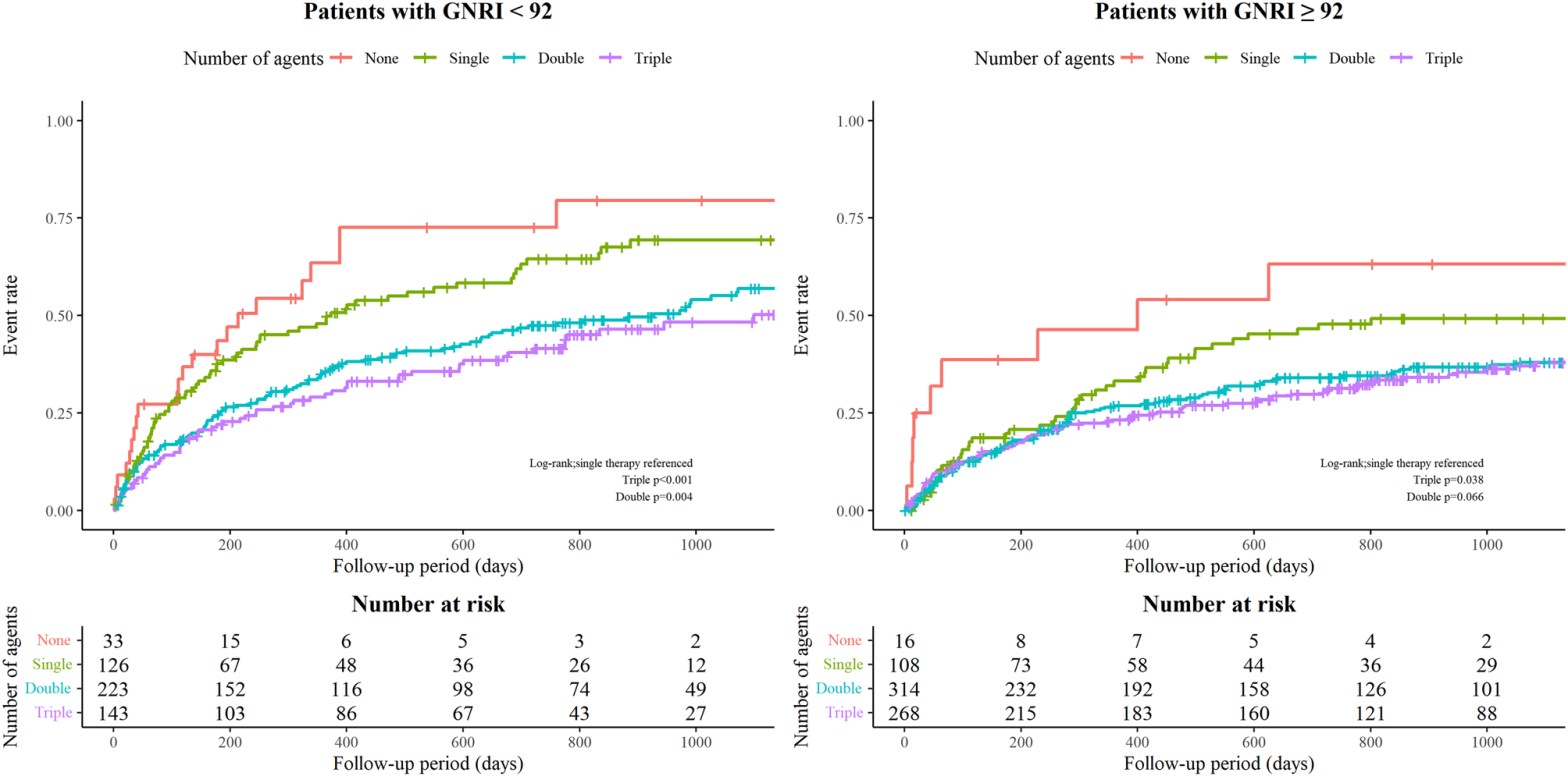
Unadjusted Kaplan–Meier curves for the composite outcome (all-cause death and heart failure rehospitalization) in each treatment group (no, single, double, and triple therapy) according to Geriatric Nutritional Risk Index score (GNRI: < 92 vs. ≥ 92).

Moreover, among the malnourished (i.e. low GNRI group), the risk for subsequent adverse outcomes was lower for patients who received double, and triple therapies than for those receiving a single agent (Fig. 4). In the low GNRI (< 92) subgroup, triple therapy (HR 0.57, 95% CI 0.40–0.82; p = 0.003) and double therapy patients (HR 0.68, 95% CI 0.50–0.94; p = 0.019) had better outcome when referenced to single therapy patients. On the contrary, within the high GNRI group, triple therapy (HR 0.95, 95% CI 0.65–1.39; p = 0.780) and double therapy patients (HR 0.74, 95% CI 0.52–1.06; p = 0.104) had no significant relationship with the composite outcome (P value for interaction also was not significant; p = 0.550). These results indicate that the advantage of combination therapy may be particularly prominent among malnourished patients rather than among those with normal nutritional status.

### Sensitivity analysis

The results of our analyses were also similar in sensitivity analyses that excluded patients with advanced renal impairment (estimated glomerular filtration rate [eGFR]: < 30 mL/min/1.73 m^2^) or higher mortality risk (Get With The Guideline-Heart Failure [GWTG-HF] risk score: > 57) (Supplementary Fig. S6). Compared with patients who received single therapy, the risk of the composite outcome was higher with those with not receiving any of the agents (adjusted HR: 1.81, 95% CI 1.10–2.96; p = 0.018) and lower in those receiving double therapy (adjusted HR: 0.66, 95% CI 0.50–0.88; p = 0.004) and triple therapy (adjusted HR: 0.69, 95% CI 0.51–0.93; p = 0.015). This tendency was consistent in the risk reduction of all-cause death (triple therapy: HR: 0.66, 95% CI 0.43–1.02; p = 0.061 or double therapy: HR: 0.55, 95% CI 0.37–0.84; p = 0.005) and HF rehospitalization (triple therapy: HR: 0.65, 95% CI 0.46–0.92; p = 0.014 or double therapy: HR: 0.67, 95% CI 0.49–0.93; p = 0.016).

Furthermore, the results of our analyses were also similar to that of the sensitivity analysis using the GNRI cutoff of 94.1 with the whole analytic cohort (Supplementary Fig. S7).

## Discussion

This study evaluated data from a contemporary multicenter registry of acute HF cases and revealed three main findings. First, among patients with HFrEF, malnutrition (based on a low GNRI score) was associated with significantly lower rates of combination medical therapy initiation. Second, patients with malnutrition had an increased risk of the composite outcome that involved all-cause death and HF rehospitalization. Third, even among patients with malnutrition, increased use of combination medical therapy was independently associated with lower risks of the composite outcome of rehospitalization or all-cause mortality.

Treatment for HF aims to prevent morbidities and prolong morbidity-free survival. Combination therapy using RAASis (i.e., ACEis/ARBs/ARNIs and MRAs) with BBs is a guideline-recommended standard therapy for patients with HFrEF. However, despite increased awareness of this recommendation, a study of the CHAMP-HF registry demonstrated that prescription rates for these disease-modifying drugs remain lower than expected in the ambulatory setting and have not improved over 15 years since the Euro Heart Survey on Heart Failure^9,28^. Similarly, a study on acute HF patients hospitalized with HFrEF from the Japanese Cardiac Registry of Heart Failure (2004–2005) revealed low proportions of drug use at discharge (BBs: 57.9%, ACEis: 4.3%, ARBs: 45.6%, MRAs: 46.0%)^29^. Aside from the rate of BB use, our findings are generally comparable and suggest that there is room for improvement in the treatment rates of ACEi/ARB and MRA, similar to that observed in Western countries. Additionally, the rate of MRA administration was similar across nutritional status. The purpose of its use may have been different than that of the other two agents and it may be prescribed as a potassium-sparing diuretic more frequently than as a cardioprotective agent. This may have influenced the relationship between its administration and GNRI value.

The gaps in combination medical therapy use in this setting may be related to various factors, including physician aversion and inertia, patient intolerance, and side effects^9^. Frailty, a possible reason for physicians being hesitant to change therapy, is also strongly related to malnutrition, but has different features that require investigation and call for further evaluation of the interaction and its association with HF treatment and outcomes, since either of the two may lead to a vicious cycle of physical impairment. In previous studies, patients with low GNRI were more likely to have characteristics of frailty, which represents a state of increased vulnerability to stressors resulting from multisystem dysregulation and is associated with a higher risk of impaired physical function and mortality^30^. As our study showed that combination medical therapy use was associated with better outcomes even in patients with low GNRI, it may be possible to improve their prognoses.

Malnutrition may make physicians reluctant to alter a patient’s treatment considering the medicine’s side-effects during liver dysfunction^16^, as it decreases oxidative metabolism in the liver, which is performed by cytochrome P-450 isoenzymes, via depletion of nicotinamide adenine dinucleotide phosphate reserves. Other liver metabolic pathways can also be impaired, which can decrease drug clearance, thereby increasing drug exposure and potentially resulting in harmful side effects. There are also concerns regarding drug absorption changes due to poor nutritional status (e.g., because of vitamin and/or mineral deficiency), which may influence the physician’s decisions based on the medication’s effectiveness and potential side effects^31^ and partially explain the present study’s findings. Moreover, the existing RCTs have generally excluded malnourished patients, who are often older and have more comorbidities. Thus, although there is still limited evidence regarding whether nutritional status influences the effectiveness and side effects of optimal medical therapies particularly when they are combined, our results suggest that even malnourished patients with HFrEF can benefit from prescription of combination medical therapy.

Malnourished patients are often older and tend to have a greater number of comorbidities, including renal dysfunction, which may make physicians hesitant to add new medications^32^. We have previously reported that RASi prescriptions were more appropriate for patients with better renal function (eGFR: > 30 mL/min/1.73 m^2^), and RASi prescription was associated with lower risks of all-cause death and HF rehospitalization, although RASi use was not significantly associated with adverse outcomes among patients with a lower eGFR^33^. Similar results have been reported for MRA use, as data from the GWTG-HF registry revealed that spironolactone was less frequently prescribed for HFrEF patients with advanced renal impairment (serum creatinine: 1.5–2.0 mg/dL) than for patients with serum creatinine concentrations of < 1.5 mg/dL^34^. Another report based on the OPTIMIZE-HF registry revealed that MRA use was associated with good clinical outcomes, even among older patients, if their eGFR was > 30 mL/min/1.73 m^2^^35^. Ferreira et al. also evaluated MRA prescription patterns in a sub-analysis of data from RCTs and reported that lower MRA doses were prescribed to patients with chronic kidney disease^36^. This may be because these patients experience more frequent episodes of worsening renal function during MRA treatment, which necessitates close surveillance and dose adaptations. Nevertheless, patients still benefited from lower MRA doses. In this clinical context, the latest European Cardiology Society Guidelines for the diagnosis and treatment of acute and chronic heart failure recommend initial combination therapies with low doses for HF with reduced ejection fraction followed by up-titration of each agent if possible^37^. Clinicians must consider the risks and benefits of any specific treatment strategy, with careful dose adjustment, and initiate combination therapy as much as possible.

### Strengths and limitations

This study highlights the real-world challenges of HF management in malnourished patients, although it also has several inherent limitations. First, the retrospective observational nature is prone to bias and confounding by unmeasured or unidentified variables, although we adjusted the analyses for relevant clinical characteristics and tested different cut-offs as sensitivity analyses. Although well-designed RCTs would be needed to ensure the efficacy of combination medical therapy, especially triple therapy, given the strength of the evidence for combination medical therapy among non-malnourished patients, there may be ethical questions regarding testing these therapies against placebo in clinical trials. In addition, the registry did not contain data regarding the specific doses of each agent and their titration or withdrawal after discharge, which precluded dose-related analyses. In the treatment of HF, titration and discontinuation of medications are not uncommon^38^, thus future studies including this information is needed for patients with malnourishment. Finally, frailty can coexist with malnutrition, although we were unable to consider the relationship between these two conditions because of the lack of data regarding frailty.

## Conclusion

Malnourished HFrEF patients, who are typically excluded from clinical trials, exhibited poorer prognoses and combination medical therapy prescription rates, when compared with patients with better nutritional status. However, our study revealed that combination medical therapy was independently associated with favorable outcomes regardless of nutritional status. Effective approaches to increase combination medical therapy utilization are needed.

## Supplementary Information

Supplementary Information.

## Data Availability

The data that support the findings of this study are available from the corresponding author, upon reasonable request.
