# Supplementary material for: Potential association with malnutrition and allocation of combination medical therapies in hospitalized heart failure patients with reduced ejection fraction
Source: Sci Rep. 2022 May 18;12:8318. doi: 10.1038/s41598-022-12357-4 (PMC9117205; doi:10.1038/s41598-022-12357-4)

Supplementary File of "Potential Association with Malnutrition and Allocation of Combination Medical Therapies in Hospitalized Heart Failure Patients with Reduced Ejection Fraction"

Authors:

Yumiko Kawakubo, MD,[a] Yasuyuki Shiraishi, MD,[a] Shun Kohsaka, MD,[a*] Takashi Kohno, MD,[b] Ayumi Goda, MD,[b] Yuji Nagatomo, MD,[c] Yosuke Nishihata, MD,[d] Mike Saji, MD,[e] Makoto Takei, MD,[f] Yukinori Ikegami, MD,[g] Nozomi Niimi MD,[a] Alexander Tarlochan Singh Sandhu, MD, [h] Shintaro Nakano, MD,[i] Tsutomu Yoshikawa, MD,[e] and Keiichi Fukuda, MD,[a] from the West Tokyo Heart Failure Registry

[a]Department of Cardiology, Keio University School of Medicine, Tokyo, Japan

[b]Department of Cardiovascular Medicine, Kyorin University Faculty of Medicine, Tokyo, Japan

[c]Department of Cardiology, National Defense Medical College Hospital, Saitama, Japan

[d]Department of Cardiology, St. Luke's International Hospital, Tokyo, Japan

[e]Department of Cardiology, Sakakibara Heart Institute, Tokyo, Japan

[f]Department of Cardiology, Saiseikai Central Hospital, Tokyo, Japan

[g]Department of Cardiology, National Hospital Organization Tokyo Medical Center, Tokyo, Japan

[h]Department of Cardiovascular Medicine, Stanford University, California, United States of America

[i]Department of Cardiology, Saitama Medical University International Medical Center, Saitama, Japan

Supplementary Figure S1. Unadjusted Kaplan-Meier survival curves for the composite outcome (all-cause death and heart failure rehospitalization) according to low and high Geriatric Nutritional Risk Index scores (GNRI: <92 vs. ≥92).

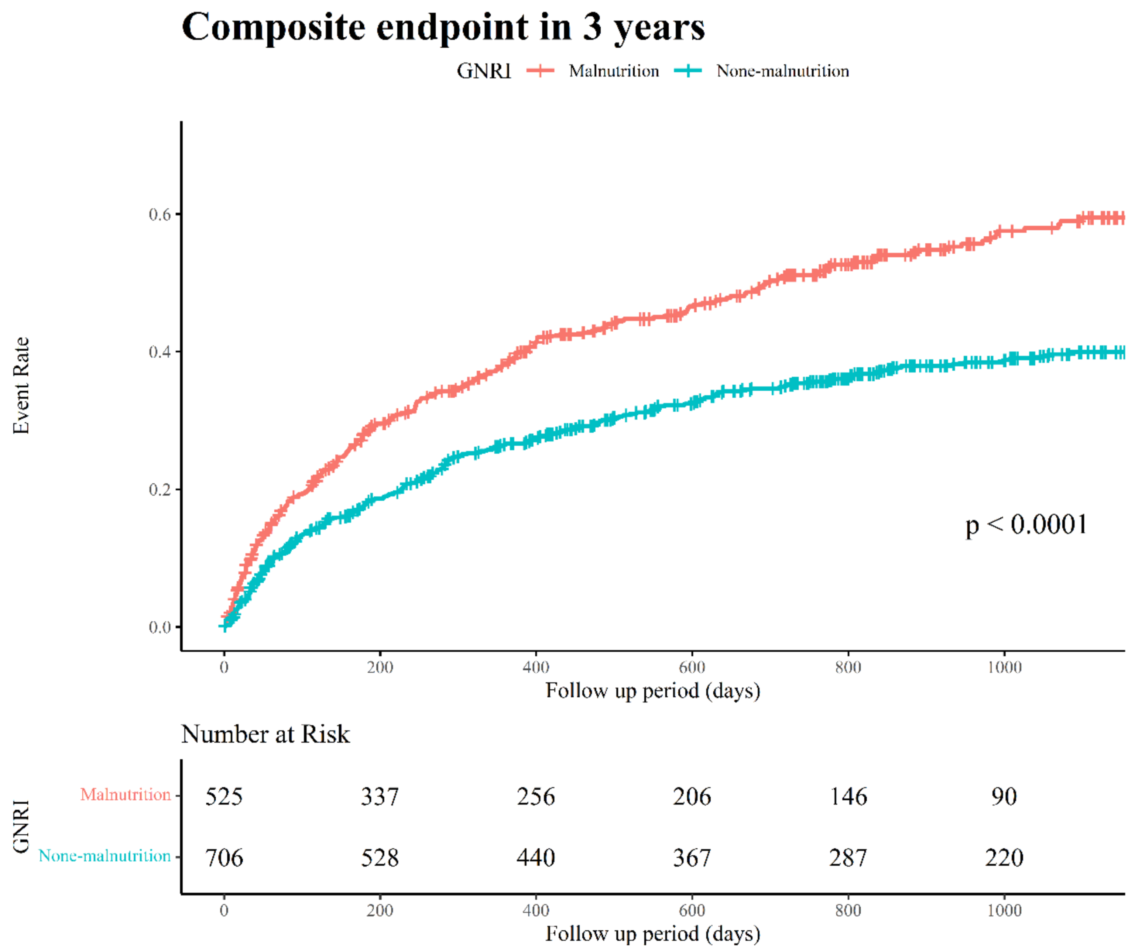

Supplementary Figure S2. Prescription rates of optimal medical therapy.

RAS inhibitor, renin-angiotensin system inhibitor; MRA, mineralocorticoid receptor antagonist

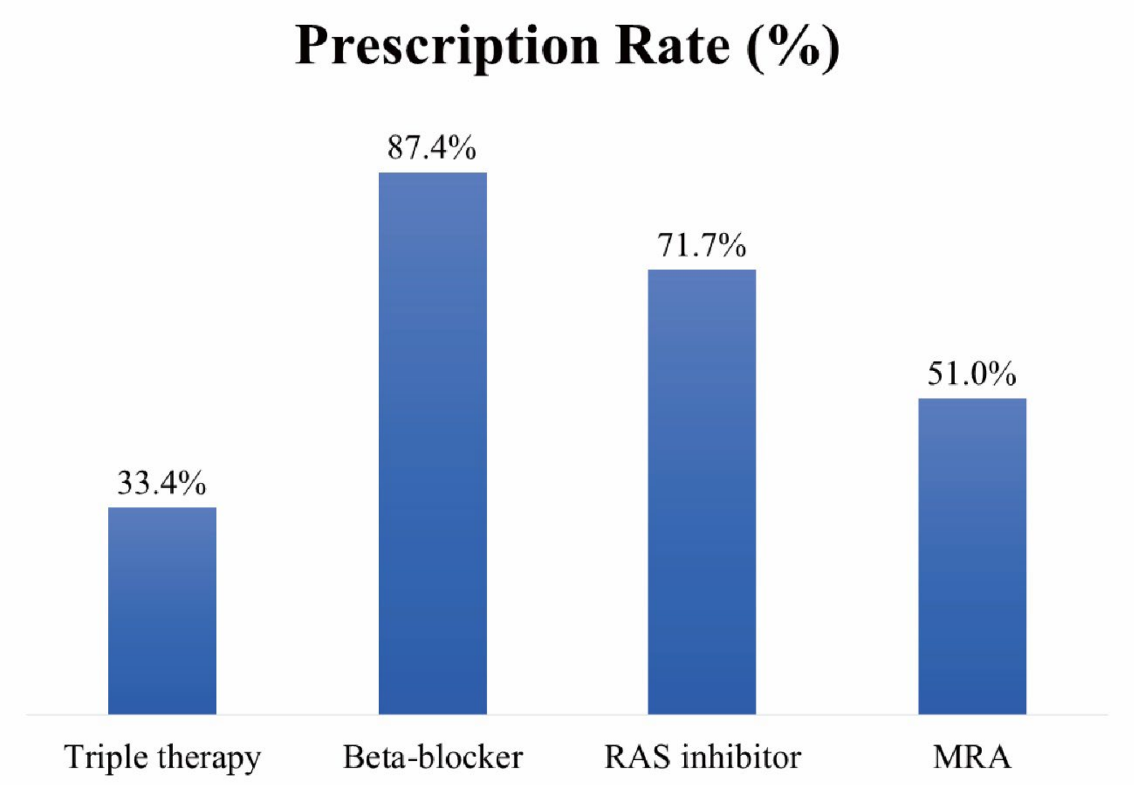

Supplementary Figure S3. Unadjusted Kaplan-Meier curves for the composite outcome (all-cause death and heart failure rehospitalization) in each treatment group (no, single, double, and triple therapy).

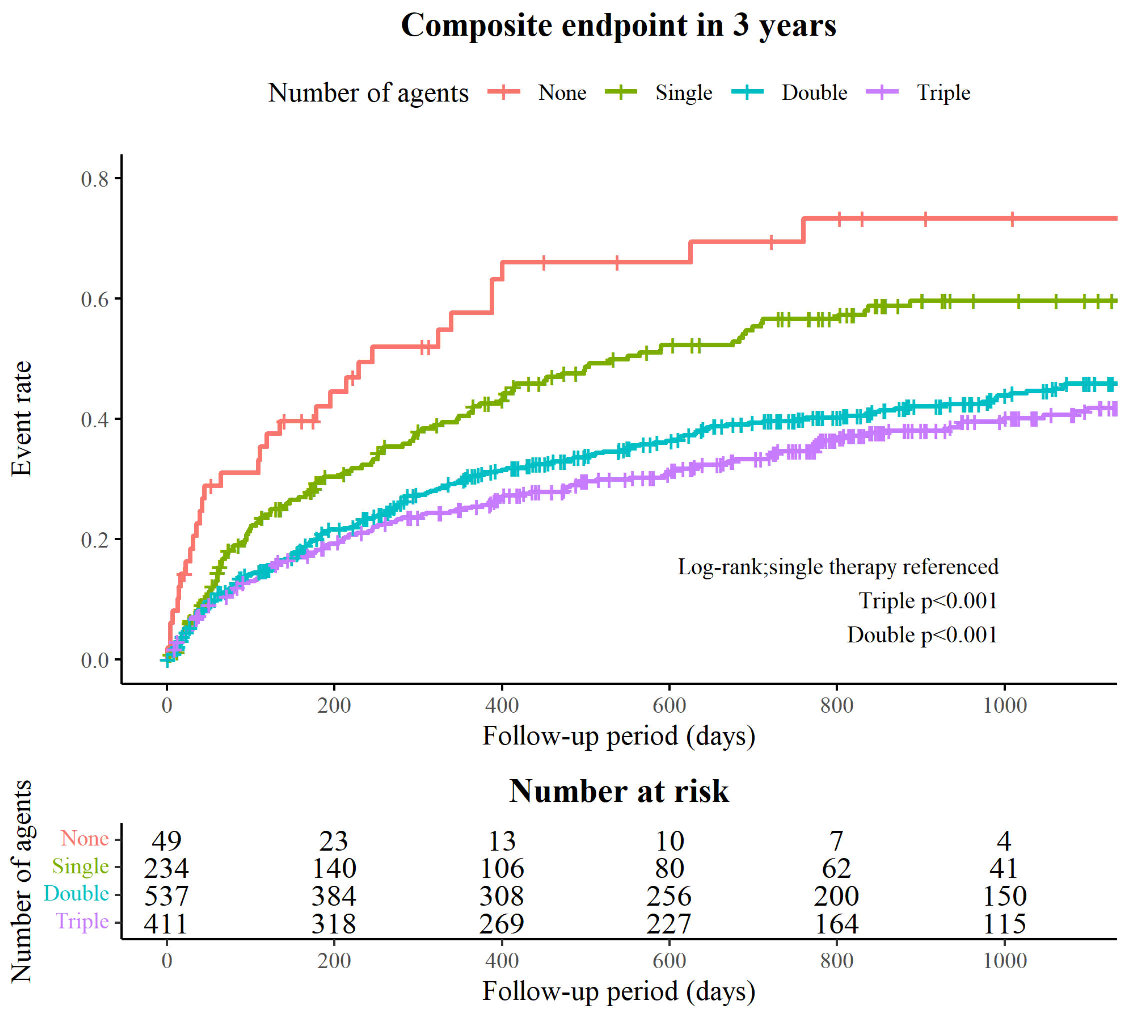

Supplementary Figure S4.

(a) Unadjusted Kaplan-Meier survival curves for all cause death according to low and high Geriatric Nutritional Risk Index scores (GNRI: <92 vs. ≥92).

(b) Unadjusted Kaplan-Meier curves for all cause death in each treatment group (no, single, double, and triple therapy).

(c) Multivariable Cox proportional hazard models of the secondary outcome; all cause death.

These models were adjusted by the following variables: age, sex, systolic blood pressure, heart rate, renal dysfunction (eGFR <60 ml/min/1.73m^2), ejection fraction, history of heart failure hospitalization, ischemic etiology, atrial fibrillation, chronic obstructive pulmonary disease, stroke, diabetes mellitus, use of loop diuretics, use of statins and medical therapy (triple, double, single, and no optimal medical therapy), as well as geriatric nutritional risk index.

(a) Death in 3 years

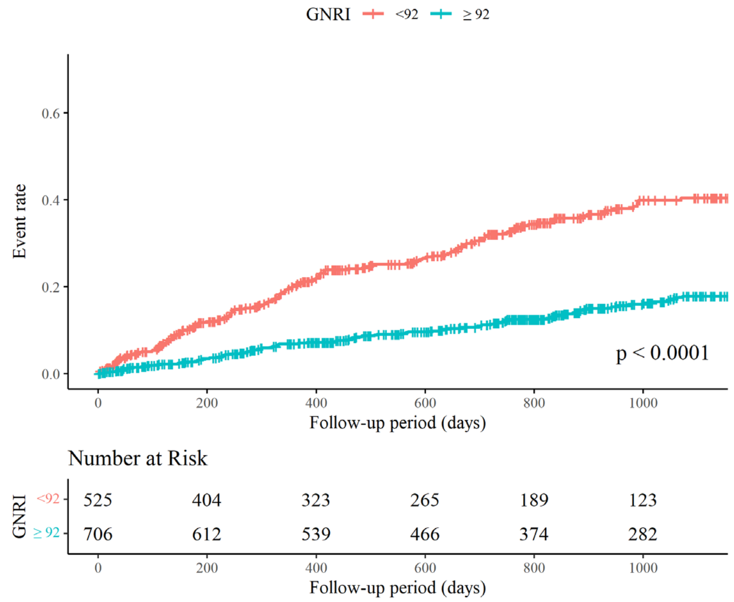

(b) Death in 3 years

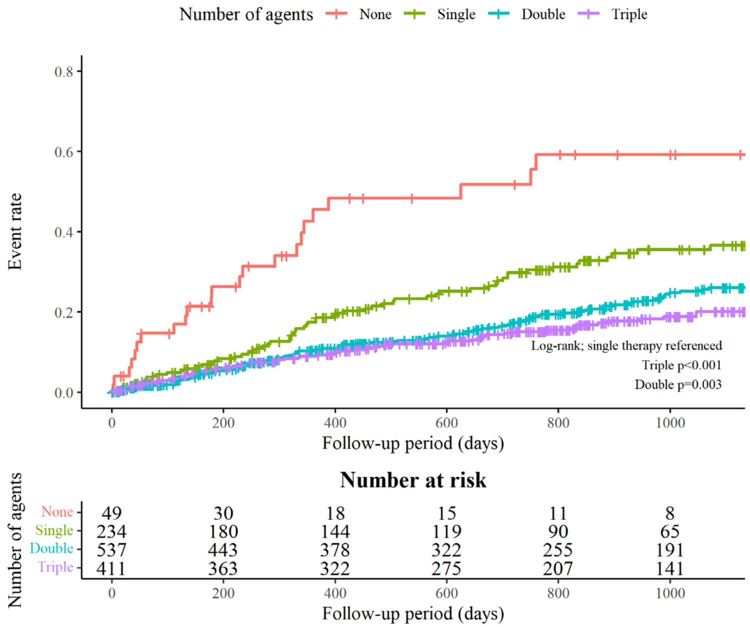

(c)

Medication class	Outcome analysis	
	**HR [95%CI]**	**p value**
Triple therapy	0.67 [0.46-0.96]	0.028
Double therapy	0.69 [0.50-0.96]	0.028
Single therapy	Reference	
None medical therapy	2.28 [1.37-3.80]	0.002

Supplementary Figure S5.

(a) Unadjusted Kaplan-Meier survival curves for HF rehospitalization according to low and high Geriatric Nutritional Risk Index scores (GNRI: <92 vs. ≥92).

(b) Unadjusted Kaplan-Meier curves for HF rehospitalization in each treatment group (no, single, double, and triple therapy).

(c) Multivariable Cox proportional hazard models of the secondary outcome; HF rehospitalization.

These models were adjusted by the following variables: age, sex, systolic blood pressure, heart rate, renal dysfunction (eGFR <60 ml/min/1.73m^2), ejection fraction, history of heart failure hospitalization, ischemic etiology, atrial fibrillation, chronic obstructive pulmonary disease, stroke, diabetes mellitus, use of loop diuretics, use of statins and medical therapy (triple, double, single, and no optimal medical therapy), as well as geriatric nutritional risk index.

(a) HF hospitalization in 3 years

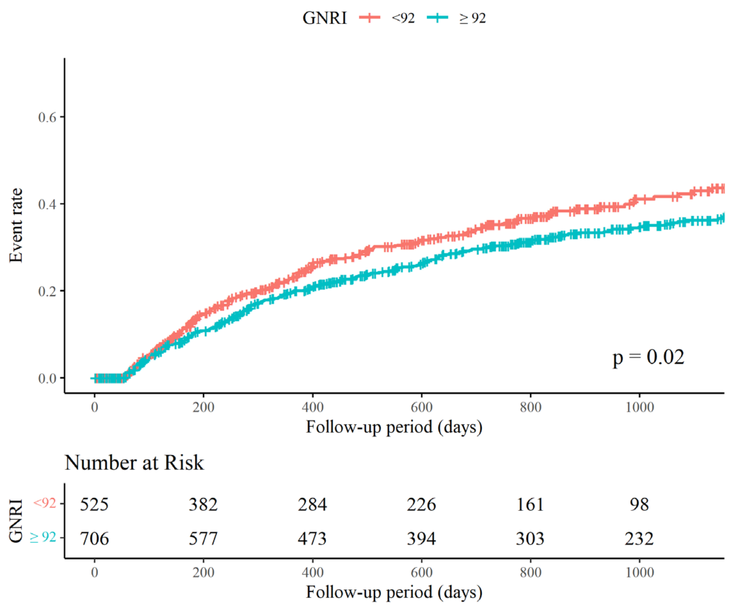

(b) HF hospitalization in 3 years

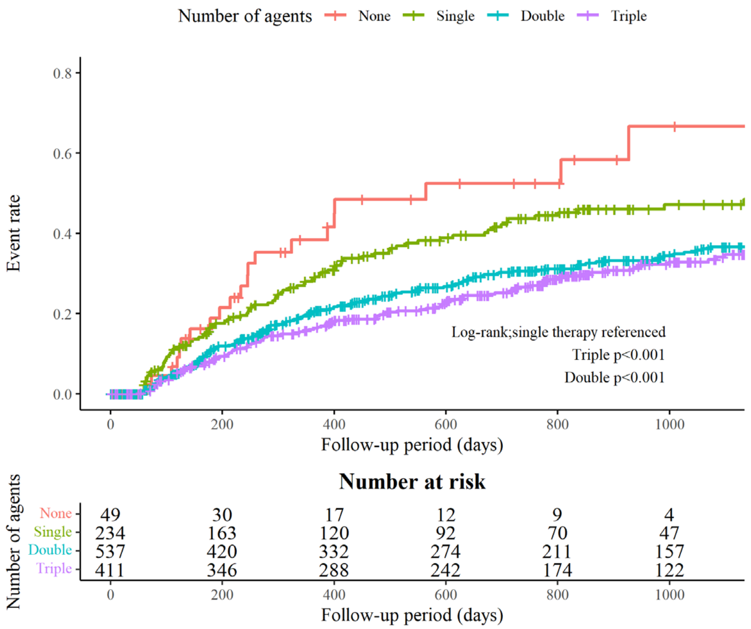

(c)

Medication class	Outcome analysis	
	HR [95%CI]	p value
Triple therapy	0.65 [0.49-0.87]	0.003
Double therapy	0.67 [0.52-0.88]	0.004
Single therapy	Reference	
None medical therapy	1.52 [0.91-2.52]	0.109

Supplementary Figure S6. Results of analyses that excluded patients with advanced renal impairment (eGFR: <30 mL/min/1.73 m2) and a higher mortality risk (Get With The Guideline-Heart Failure risk score: >57).

(a) Unadjusted Kaplan-Meier curves for the composite outcome (all-cause death and heart failure rehospitalization) according to low and high Geriatric Nutritional Risk Index scores (GNRI: <92 vs. ≥92).

(b) Unadjusted Kaplan-Meier curves for the composite outcome (all-cause death and heart failure rehospitalization) in each treatment group (no, single, double, and triple therapy) according to Geriatric Nutritional Risk Index score (GNRI: <92 vs. ≥92).

(c) Multivariable Cox proportional hazard models of the composite outcome (all-cause death and heart failure rehospitalization).

These models were adjusted by the following variables: age, sex, systolic blood pressure, heart rate, renal dysfunction (eGFR <60 ml/min/1.73m^2), ejection fraction, history of heart failure hospitalization, ischemic etiology, atrial fibrillation, chronic obstructive pulmonary disease, stroke, diabetes mellitus, use of loop diuretics, use of statins and medical therapy (triple, double, single, and no optimal medical therapy), as well as geriatric nutritional risk index.

(a) Composite endpoint in 3 years

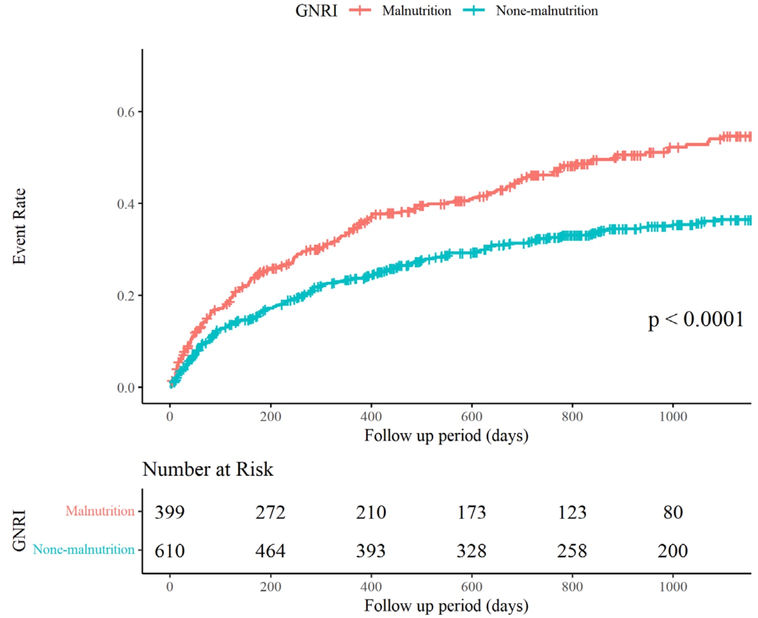

(c)

Medication class	Outcome analysis	
	HR [95%CI]	p value
Triple therapy	0.69 [0.51-0.93]	0.015
Double therapy	0.66 [0.50-0.88]	0.004
Single therapy	Reference	
None medical therapy	1.81 [1.10-2.96]	0.018

(b)

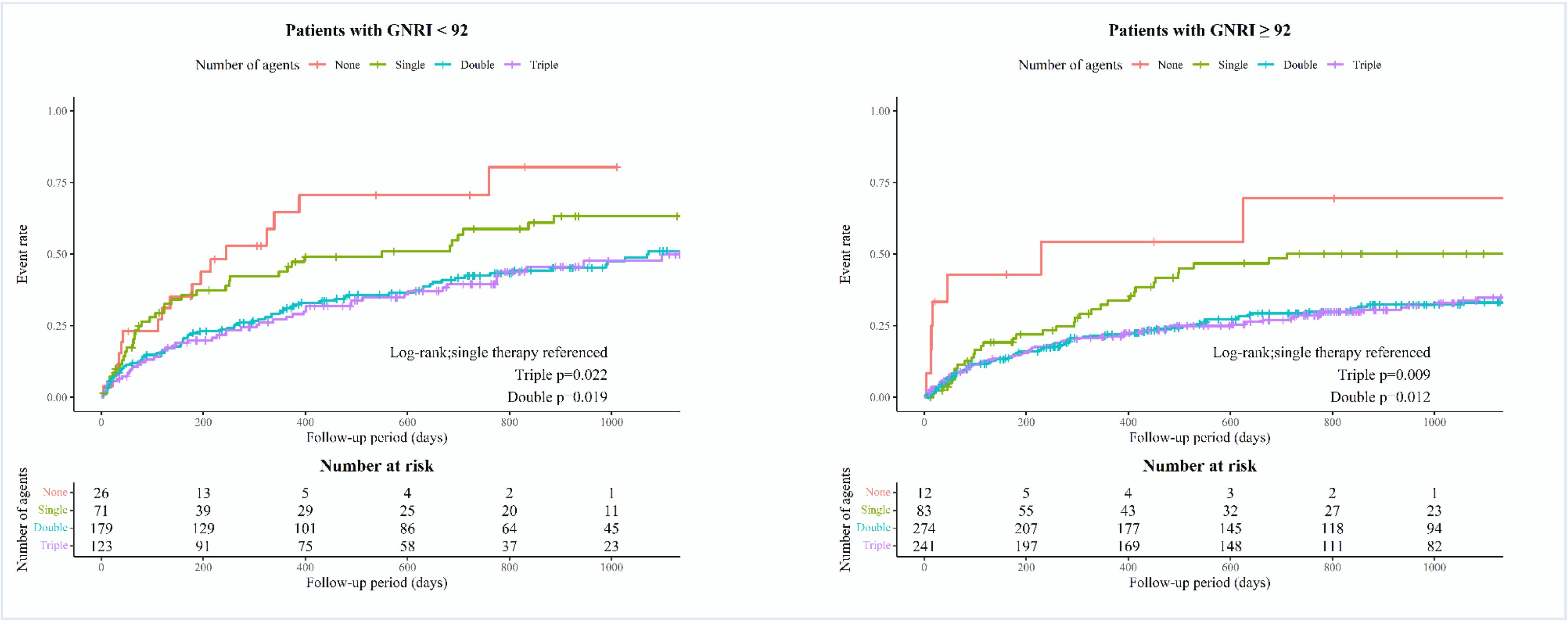

Supplementary Figure S7.

(a) Unadjusted Kaplan-Meier survival curves for the composite outcome (all-cause death and heart failure rehospitalization) according to low and high Geriatric Nutritional Risk Index scores (GNRI: <median vs. ≥median).

(b) Unadjusted Kaplan-Meier curves for the composite outcome (all-cause death and heart failure rehospitalization) in each treatment group (no, single, double, and triple therapy) according to Geriatric Nutritional Risk Index score (GNRI: <median vs. ≥median)

(a) Composite endpoint in 3 years

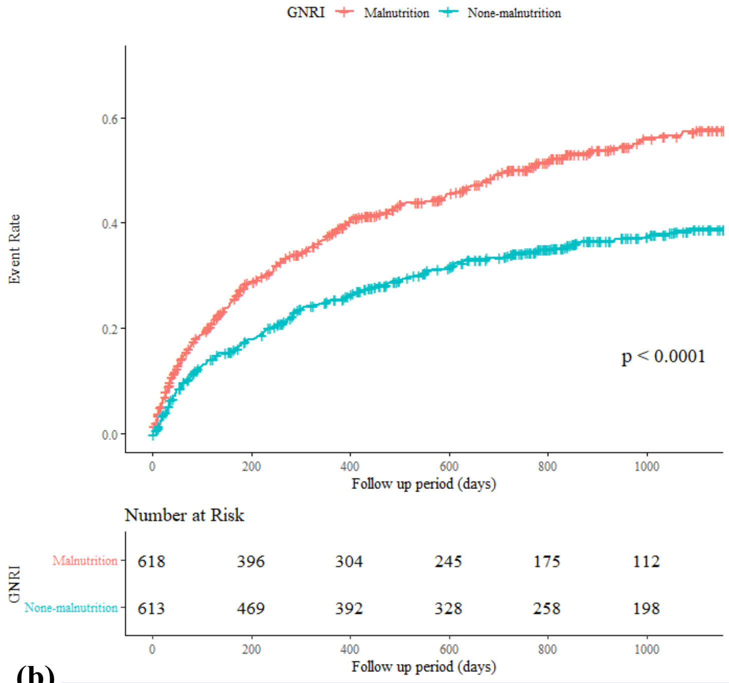

(b)

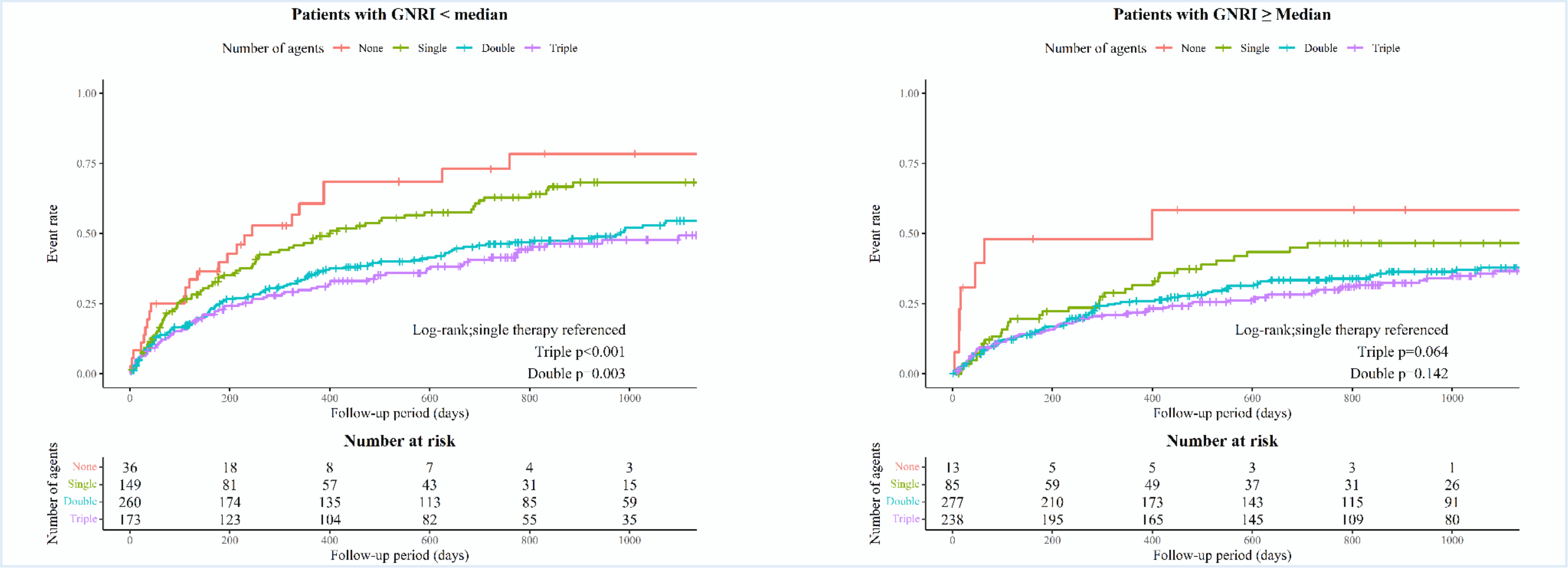

Supplement: Supplementary file 1 — Supplementary Information. [file 41598_2022_12357_MOESM1_ESM.pdf]
